# The Relationship between Physical Activity, Sleep Quality, and Stress: A Study of Teachers during the COVID-19 Pandemic

**DOI:** 10.3390/ijerph192315465

**Published:** 2022-11-22

**Authors:** Fabio Fontana, Kelsey Bourbeau, Terence Moriarty, Michael Pereira da Silva

**Affiliations:** 1 Department of Kinesiology, University of Northern Iowa, Cedar Falls, IA 50614, USA; 2 Faculty of Medicine, Federal University of Rio Grande, Rio Grande 90040-060, Brazil

**Keywords:** COVID-19, teacher, mediating effect, physical activity, sleep, stress

## Abstract

The COVID-19 pandemic prompted chaotic changes in the daily lives of K-12 teachers, resulting in increased stress and other mental health problems. Limited evidence regarding the relationship between physical activity, sleep, and perceived stress among teachers during the COVID-19 pandemic exists. Therefore, the purpose of this study was to investigate the association between physical activity, sleep quality, and perceived psychological stress among teachers during the COVID-19 pandemic. An online survey measuring physical activity, sleep quality, and perceived psychological stress was distributed across 47 US states between September and October of 2020. Data provided by 635 teachers (mean age: 42 ± 18 years, 74.6% female) were included in the present analysis. Results suggested a negative association between physical activity and perceived psychological stress. Mediation analyses indicated that teachers engaging in high levels of physical activity were more likely to have good quality sleep and, in turn, were less likely to report high levels of perceived stress. Physical activity and sleep-related interventions could help curtail the rising levels of psychological distress amongst K-12 teachers during stressful times such as the COVID-19 pandemic. Teachers, especially those that self-report as female and/or young, report high levels of stress. These high levels of stress are a serious challenge for school districts in terms of attracting and retaining qualified teachers in K-12 classrooms.

## 1. Introduction

The coronavirus pandemic upended societies globally. At the beginning of the pandemic, state governments in the US declared a state of emergency and implemented policies to reduce the rate of transmission of COVID-19, such as banning of large social gatherings, mask mandates, and the closure of exercise and entertainment establishments. Simultaneously, school districts responded by moving instruction to a remote mode. A stressful profession in normal times [1,2,3], switching to distance education created its unique set of stressors for teachers, such as unfamiliarity with online education, concerns about the quality of online learning experiences especially for students in more vulnerable groups (e.g., low socioeconomic status), and difficulties balancing work with family life [4,5,6,7,8,9,10,11]. A substantial proportion of teachers reported high levels of perceived stress as K-12 education moved online [4,5].

As the pandemic progressed, some school districts in the US returned to in-person learning in the fall 2020. Although the pandemic was still raging in the US, it became clear that in-person learning was fundamental to the academic, physical, and psychological development of children [12,13,14,15,16,17,18]. School districts returning to in-person instruction implemented safety measures to mitigate the risk of COVID-19 spread, such as increased ventilation, social distancing, body temperature checks, masking, and desk shields. Adapting to the COVID-19 safety protocols presented its own challenges for teachers. For example, masking muffles the voice and muddles the reading of facial expressions by students and teachers. Due to increased interactions with students and peers during in-person instructions, teachers also reported concerns about their health and the health of their families and communities [5]. The coronavirus pandemic added significant stressors to the teaching profession even for schools that reopened for in-person learning. In fact, 19% of teachers in Spain reported severe levels of stress as they returned to in-person instruction in the fall of 2020 [19].

Lifestyle behaviors such as physical activity (PA) and sleep are possible preventive strategies to manage stress. For example, multiple investigations have reported a negative association of PA with stress and professional burnout [20,21]. Physical activity has also been shown to buffer the effects of stress on physical and mental-health-related outcomes during the academic examination period of college students [22]. Conversely, shorter sleep duration and lower sleep quality are associated with increases in the levels of perceived stress [23,24,25,26]. These relationships have also been evidenced during the COVID-19 pandemic. Higher engagement in PA and longer sleep duration were associated with lower levels of perceived levels of stress during the pandemic [27,28]. More importantly, avoiding a reduction in PA and sleeping duration may confer some protection against increases in the level of pandemic-related perceived stress [20,28]. Finally, more engagement in PA is associated with longer sleep duration and improved sleep quality [29,30]. Although the body of evidence indicating a relationship between stress, PA, and sleep quality is robust, investigations conducted during the pandemic focused mostly on college students and healthcare professionals. In addition to focusing on teachers, this investigation examined a particular period during the COVID-19 pandemic when US schools were transitioning from a state of complete lockdown to a gradual return to in-person activities. Thus, the purpose of this study was to further examine the association between PA, sleep quality, and perceived psychological stress among teachers during the COVID-19 pandemic. Existing literature suggests that PA is negatively associated with perceived psychological stress during the pandemic. Moreover, PA may indirectly improve stress through its positive impact on sleep quality. We hypothesized that sleep quality will mediate the relationship between PA and perceived psychological stress. Describing the levels of perceived stress by sex and age is relevant considering that teaching, a female-dominated profession, has faced difficulties attracting young professionals even before the onset of the pandemic [31]. In line with previous evidence [28], we also hypothesized that women and younger professionals will report higher levels of perceived stress.

## 2. Materials and Methods

Non-probability purposive sampling was used to select currently employed elementary, middle school, and high school teachers from public school districts across 47 states in the United States during the months of September and October, 2020. Public school district information (number of students, schools in district, size of schools) was obtained using the U.S. Department of Education search tool on the National Center for Education Statistics website. The largest school district from the largest county from each state was selected. Further, the high school, middle school, and elementary school with the highest number of students within the selected school district was chosen as the sample. If a selected county or school district did not provide publicly available email addresses on their website, the next largest county or school district was selected. All teachers listed on the school websites were emailed a link to an anonymous Qualtrics survey that contained a series of questionnaires. Teachers from North Dakota, Virginia, and Wyoming were not included in the present study, as no publicly available email addresses could be located. The number of students in each school district ranged from 1522 to 495,255 students. A total of 9846 teachers were invited to participate in the study for a response rate of 8.4%. All questionnaires were administered remotely, online via Qualtrics (Qualtrics Corp., Drive Provo, UT, USA). The study was approved by the institutional review board (approval number 21-0011) and participants consented prior to data collection.

### 2.1. Instrumentation

#### 2.1.1. Perceived Stress Scale

The Perceived Stress Scale (PSS-10) is a self-report measure of perceived stress in adults [32]. The scale consisted of 10 items asking about feelings of stress experienced in the past month (e.g., “In the last month, how often have you felt confident about your ability to handle your personal problems?”). Each item was scored on a five-point Likert scale ranging from 0 (never) to 4 (very often). Items four, five, seven, and eight were reverse coded. Total scores ranged from 0 to 40, with higher scores reflecting higher levels of perceived stress. Scores between 0–13, 14–26, and 27–40 were considered low, moderate, and high levels of perceived stress, respectively [33]. Extensive and strong evidence indicates that the PSS-10 is a valid and reliable measure of perceived stress in adults 31–34 (e.g., internal consistency Cronbach’s alpha and test–retest reliability scores were >0.70 and strong correlations with other measures of mental health were displayed). 

#### 2.1.2. International Physical Activity Questionnaire-Short Form

The International Physical Activity Questionnaire-Short Form (IPAQ-SF) is a six-item self-report measure of the PA performed in the past seven days [34]. The items ask questions about the amount of time and days respondents engaged in vigorous, moderate, or walking types of PA. Prior evidence indicates that the IPAQ-SF is a valid and reliable measure of PA in adults [34,35] (e.g., test–retest ICC = 0.76–0.86, 80% agreement between IPAQ-SF and accelerometry, and significant correlation between IPAQ-SF and accelerometry).

#### 2.1.3. Short Pittsburgh Sleep Quality Index

The Short Pittsburgh Sleep Quality Index (shortPSQI) is a self-report measure of sleep quality in adults [36]. The scale is adapted version of the original, commonly used, and extensively validated Pittsburgh Sleep Quality Index [37]. The shortPSQI consisted of 13 items grouped into five components: sleep duration, sleep latency, sleep efficiency, sleep disturbances, and daytime dysfunction. Each component of the shortPSQI was scored between 0 and 3 points. Global scores for the shortPSQI ranged between 0 and 15, and a score above 4 indicated a poor-quality sleeper [36]. Global scores and sleep quality classifications (i.e., good or poor sleeper) between the original and short PSQI surveys were strongly correlated. Prior evidence indicates that the shortPSQI is a valid and reliable measure of sleep quality in adults [36,37] (e.g., internal consistency Cronbach’s alpha of PSQI and test–retest reliability scores were >0.70, there were significant correlations with polysomnographic measures of sleep, and, finally, the shortPSQI was strongly correlated with PSQI and had adequate diagnostic sensitivity and specificity scores to distinguish between good and poor sleepers). 

### 2.2. Data Analysis

Descriptive statistics (median, interquartile range (IQR), frequencies, and percentages) were computed to examine the characteristics of participants. Differences between genders were tested using the Mann–Whitney test for numerical variables (age, years teaching, PSQ Global Score, Total PA, and PSS scores) and the chi-square test for categorical variables (race, sleep quality, and stress level). Crude and adjusted ordinal regression analyzed the association of age, gender, race, teaching level, teaching method, years teaching, sleep quality, and PA level (based on tertiles of overall PA represented in minutes/week) with stress levels (low, moderate, and high). Only variables with *p* < 0.20 were kept in the final adjusted model. Mediation analysis was carried out through path analysis using a generalized structural equation model (GSEM) based on an ordinal logistic regression. We tested the model fit using the chi-square test (X^2^) for the likelihood ratio test, and Akaike’s information criterion (AIC), and Bayesian information criterion (BIC) to identify if the mediation models were better than the model without the mediation paths. Results from the multivariate ordinal logistic regression and the mediation analysis were expressed in crude and adjusted odds ratio (AOR) and 95% confidence intervals (95%CI). The significance level was set at *p* < 0.05 for a two-tailed test, and all analyses were performed using the Stata MP 14.1 (StataCorp LLC., College Station, TX, USA) software.

## 3. Results

A total of 831 teachers completed the Qualtrics survey. Of the 831 teachers, 196 were excluded from the analysis due to missing or incorrect data on any of the questionnaires (Gender = 4; IPAQ = 131; PSQ = 61). Thus, a total of 635 teachers were included in the present analysis. Table 1 highlights the descriptive statistics for the participants. Participants were mostly women (74.6%), white (86.8%), taught at high school level (57.1%), were teaching online (53.4%), practiced less than 150 min of moderate to vigorous physical activity per week (52.6%), with poor sleep quality (71.5%), and high levels of perceived stress (61.4%). Results indicate a higher prevalence of women teaching at the elementary level and a higher prevalence of men teaching at the high school level. Besides this, women had higher scores and prevalence of poor sleep quality and levels of perceived stress than men. 

**Figure 1:**
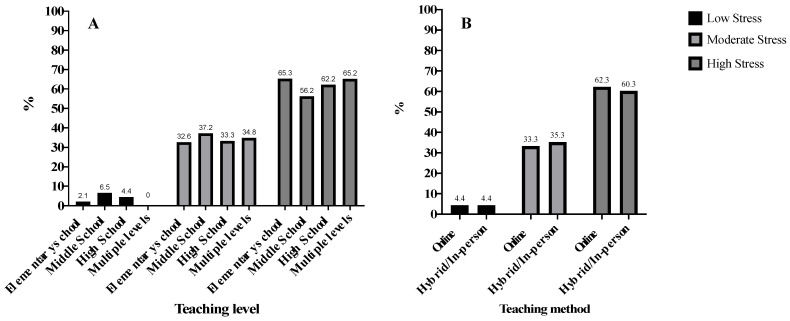
Stress level according to the teaching level (**A**) and the teaching method (**B**) (*n* = 635).

Figure 1 shows the levels of perceived stress for each teaching level and method. There were no differences in levels of perceived stress between the teaching levels (X^2^(6) = 5.26, *p* = 0.510) and the teaching methods (X^2^(2) = 0.278, *p* = 0.870). There was no significant difference in the prevalence of the teaching method across the teaching levels (Online teaching: Elementary school = 61,0%; Middle school = 52.9%; High school = 53.6%; Multiple levels = 34.8%; X^2^(3) = 5.40, *p* = 0.144). 

Table 2 shows the results of a multivariate ordinal regression analyzing the factors associated with stress levels. Older participants (AOR = 0.96; 95%CI = 0.94; 0.98), with good sleep quality (AOR = 0.24; 95%CI = 0.17; 0.35) and higher PA levels (AOR = 0.71; 95%CI = 0.57; 0.87) were less likely to report higher levels of perceived stress. Women (AOR = 2.19; 95%CI = 1.51; 3.17) were more likely to report higher levels of perceived stress.

**Figure 2:**
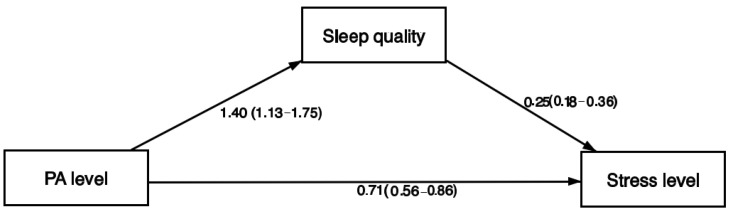
Mediation effect of sleep quality in the association of PA levels and stress level. PA level: physical activity (0 = low physical activity; 1 = moderate physical activity; 2 = high physical activity). Sleep quality: 0 = Poor; 1 = Good. Stress level: Low stress = PSS Scores < 14; Moderate stress = PSS Scores 15 to 26; High stress = PSS scores ≥ 27. Mediation analysis adjusted for age, gender, race, and years teaching. Results expressed in odds ratio and 95% confidence intervals.

We further tested the mediation of the sleep quality in the association between PA level and perceived stress level adjusted for age, gender, race, and years teaching. We found a direct effect of PA engagement on perceived stress levels (OR = 0.71; 95%CI = 0.56–0.86; *p* < 0.001); however, this association was partially mediated by sleep quality (indirect effects: OR = 0.62; 95%CI = 0.42–0.82; *p* < 0.001; mediation proportion = 72.1%). The mediation model showed a good fit (X^2^ = 55.9, *p* < 0.001) and lower AIC (1684.8) and BIC (1729.3) than the model without a mediation path (AIC = 1738.8; BIC = 1778.8; Figure 2).

## 4. Discussion

The main purpose of this study was to examine the role of sleep quality as a mediator of the relationship between PA and perceived psychological stress among teachers during the COVID-19 pandemic. Physical activity was indirectly associated with perceived psychological stress during the pandemic. Teachers engaging in high levels of PA were 29% less likely to report high levels of perceived stress. Higher participation in PA has been demonstrated as a protective factor against pandemic-related psychological stress in previous investigations [27,28]. Furthermore, sleep quality partially mediated the relationship between PA and perceived stress, explaining 72.1% of the relationship between these two variables. Fit indices indicated a better fit for the model with the mediation pathway in comparison to the model without a mediation pathway. Teachers engaging in high levels of PA were 40% more likely to have good quality sleep, and in turn teachers who had good quality sleep were 75% less likely to report high levels of perceived stress. Similar relationships have also been demonstrated in prior investigations [23,26,29]. Werneck [38] found that a worsening quality of sleep mediated the relationship between physical inactivity and incidence of mental health indicators such as loneliness, depression, and anxiety. Schools should encourage teachers to adopt healthy lifestyle behaviors such as engaging in PA and sleep in order to manage stress. School-based comprehensive PA programs, successful in promoting PA among children and adolescent students, could add a workplace component to support the engagement of teachers in PA during the school day [39]. Effective principles derived from general worksite interventions such as tailoring PA interventions to individual interests and delivering group exercise sessions can be used to increase the effectiveness of PA interventions for teachers [40].

The COVID-19 pandemic has exacerbated the typically high levels of stress associated with the teaching profession. In this study, 61.4% of the teachers reported high levels of stress. Although this is likely due to methodological differences and the country of origin of the sample of the participating teachers, it is important to note that this percentage was higher than those found in other investigations conducted during the COVID-19 pandemic [5,8,10,19]. Poor sleep compounded the high levels of stress reported by teachers, with 71.5% of the participants being classified as poor sleepers. A high level of stress among teachers is a serious health concern, not only because of its potentially negative impact on mental health, but also because teaching effectiveness may be compromised [41]. This study also showed that women and young teachers were particularly vulnerable to high levels of perceived stress during the pandemic. The teaching profession, a female-dominated profession, was experiencing a shrinkage in the pipeline of young teachers entering the field even before the pandemic started [31]. Policies to incentivize, develop, and better support teachers, including higher pay and better training, may assist in hiring and retaining talented young and mostly female teachers.

Teachers reported high levels of perceived stress irrespective of whether they returned to in-person teaching, adopted a hybrid model of teaching, or continued with online instruction during the 2020 Fall Semester. This is not surprising, considering that stressors may differ but the pandemic imposed challenges regardless of the teaching format [4,5,19]. School administrators are advised to maintain an open line of communication with teachers about their levels of perceived stress and the support necessary for effective classroom instruction. 

This study has several limitations. We used self-report surveys to collect information about sleep quality, perceived psychological stress, and PA engagement. Although the selected instruments were extensively validated, self-report measures are dependent on accurate recall and susceptible to response bias. This study was cross-sectional, which prevents causal inferences. This study did not examine changes in the perceived level of stress, physical activity, and sleep quality in comparison to levels prior to the onset of the COVID-19 pandemic. Given the response rate of the current study, care should be taken to avoid the overgeneralization of the findings in this population. Finally, the sample of participants consisted of teachers working in the largest school districts in each US state. The pandemic may have affected teachers in small or medium sized school districts differently. 

## 5. Conclusions

In this study, teachers showed high levels of stress. The findings also suggest that physical activity may improve sleep quality and reduce stress in K-12 teachers. These findings should be strongly considered when developing and implementing wellness policies and programs for teachers to improve their quality of life and their mental health, and to ensure a constant flow of high-quality teachers to teach our ever-expanding youth population. 

## Figures and Tables

**Table 1:** Descriptive characteristics of the sample (*n* = 635).

	Overall (*n* = 635)	Men (*n* = 161)	Women (*n* = 474)	*p*
Age (median (IQR))	42.0 (18.0)	42.0 (20.0)	43.0 (18.0)	0.53 ^a^
Years teaching (median (IQR))	14.0 (15.0)	14.0 (15.0)	14.0 (15.0)	0.73 ^a^
PSQ Global Score (median (IQR))	6.0 (5.0)	5.0 (3.0)	7.0 (5.0)	<0.001 ^a^
Total PA (median (IQR))	270.0 (405)	300.0 (438.0)	260.0 (380.0)	0.06 ^a^
PSS score (median (IQR))	28.0 (7.0)	26.0 (8.0)	29.0 (7.0)	<0.001 ^a^
	***n* (%)**	***n* (%)**	***n* (%)**	***p*** ^b^
**Gender**				
Men	161 (25.4)	-	-	
Women	474 (74.6)	-	-	
**Race**				
White	551(86.8)	133 (82.6)	418 (88.2)	0.19
Black or African American	28 (4.4)	9 (5.6)	10 (4.0)	
Others ^c^	56 (8.8)	19 (11.8)	37 (7.8)	
**Teaching level (*n* = 631)**				
Elementary school	95 (15.1)	7 (4.4)	88 (18.7)	<0.001
Middle school	153 (24.2)	28 (17.5)	125 (26.5)	
High school	360 (57.1)	114 (71.2)	246 (52.2)	
Multiple levels	23 (3.6)	11 (6.7)	12 (2.6)	
**Teaching method**				
Online	342 (53.4)	85 (52.8)	257 (54.2)	0.754
Hybrid or in-person	293 (46.1)	76 (47.2)	217 (45.8)	
**PA level (MVPA)** ^d^				
<150 min/week	334 (52.6)	75 (46.6)	259 (54.6)	0.07
≥150 min/week	301 (47.4)	86 (53.4)	215 (45.4)	
**Sleep quality**				
Poor	454 (71.5)	105 (65.2)	349 (73.6)	0.04
Good	181 (28.5)	56 (34.8)	125 (26.4)	
**Stress Level** ^e^				
Low	28 (4.4)	10 (6.2)	18 (3.8)	<0.001
Moderate	217 (34.2)	79 (49.1) *	138 (29.1) *	
High	390 (61.4)	72 (44.7) *	318 (67.1) *	

^a^ = *p*-value for Mann–Whitney test. ^b^ = *p*-value for chi-square test. ^c^ = Includes: American Indian or Alaska Native; Asian; Native Hawaiian or Pacific Islander; Others. ^d^ = PA level refers to reaching at least 150 min of moderate to vigorous physical activity (MVPA) per week ^e^ = Low stress: PSS Scores ≤ 14; Moderate stress: PSS Scores 15 to 26; High stress: PSS scores ≥ 27. * = significant gender differences.

**Table 2:** Crude and adjusted odds ratio (OR) and 95% confidence intervals (95%CI) for factors associated with stress levels in North American teachers (*n* = 635).

	Perceived Stress Level ^a^			
	OR	*p*	AOR (95%CI)	*p*
**Age (Years)**	0.98 (0.97; 0.99)	0.005	0.96 (0.94; 0.98)	0.001
**Gender**				
Male	Ref.		Ref.	
Female	2.41 (1.69; 3.44)		2.19 (1.51; 3.17)	<0.001
**Race**				
White	Ref.		Ref.	
Black or African American	0.46 (0.21; 0.98)	0.043	0.49 (0.22; 1.08)	0.078
Others ^b^	1.01 (0.58; 1.75)	0.980	1.04 (0.58; 1.89)	0.883
**Teaching level (*n* = 631)**				
Elementary school	Ref.		-	
Middle school	0.65 (0.39;1.10)	0.111	-	
High school	0.85 (0.53; 1.36)	0.505	-	
Multiple levels	1.02 (0.40; 2.36)	0.952	-	
**Teaching method**				
Online	Ref.		-	
Hybrid or in-person	0.93 (0.67; 1.27)	0.645	-	
**Years teaching**	0.98 (0.96; 1.00)	0.089	1.02 (0.99; 1.05)	0.098
**Sleep quality**				
Poor	Ref.		Ref.	
Good	0.24 (0.17; 0.35)	<0.001	0.24 (0.17; 0.35)	<0.001
**PA level ^c^**	0.66 (0.54; 0.81)	<0.001	0.71 (0.57; 0.87)	0.001

^a^: Low stress: PSS scores ≤ 14; moderate stress: PSS scores 15 to 26; high stress: PSS scores ≥ 27. ^b^: Includes: American Indian or Alaska Native; Asian; Native Hawaiian or Pacific Islander; Others. ^c^: Physical activity levels based on tertiles of the overall physical activity (minutes/week): moderate + vigorous + walking (0 = low physical activity; 1 = moderate physical activity; 2 = high physical activity). AOR: adjusted odds ratio. Adjusted model only kept the variables with *p* < 0.20).

## Data Availability

The group data presented in this study are available on request from the corresponding author. The individual data are not publicly available due to privacy and confidentiality.

## References

[1] Agai–Demjaha T., Minov J., Stoleski S., Zafirova B. (2015). Stress Causing Factors among Teachers in Elementary Schools and Their Relationship with Demographic and Job Characteristics. Open Access Maced. J. Med. Sci..

[2] Bottiani J.H., Duran C.A.K., Pas E.T., Bradshaw C.P. (2019). Teacher Stress and Burnout in Urban Middle Schools: Associations with Job Demands, Resources, and Effective Classroom Practices. J. Sch. Psychol..

[3] Kourmousi N., Alexopoulos E.C. (2016). Stress Sources and Manifestations in a Nationwide Sample of Pre-Primary, Primary, and Secondary Educators in Greece. Front. Public Health.

[4] Amri A., Abidli Z., Elhamzaoui M., Bouzaboul M., Rabea Z., Ahami A.O.T. (2020). Assessment of Burnout among Primary Teachers in Confinement during the COVID-19 Period in Morocco: Case of the Kenitra. Pan Afr. Med. J..

[5] Santamaría M.D., Mondragon N.I., Santxo N.B., Ozamiz-Etxebarria N. (2021). Teacher Stress, Anxiety and Depression at the Beginning of the Academic Year during the COVID-19 Pandemic. Glob. Ment. Health.

[6] Hong X., Liu Q., Zhang M. (2021). Dual Stressors and Female Pre-School Teachers’ Job Satisfaction during the COVID-19: The Mediation of Work-Family Conflict. Front. Psychol..

[7] Fan C., Fu P., Li X., Li M., Zhu M. (2021). Trauma Exposure and the PTSD Symptoms of College Teachers during the Peak of the COVID-19 Outbreak. Stress Health.

[8] Estrada-Muñoz C., Vega-Muñoz A., Castillo D., Müller-Pérez S., Boada-Grau J. (2021). Technostress of Chilean Teachers in the Context of the COVID-19 Pandemic and Teleworking. Int. J. Environ. Res. Public Health.

[9] Lizana P.A., Vega-Fernadez G., Gomez-Bruton A., Leyton B., Lera L. (2021). Impact of the COVID-19 Pandemic on Teacher Quality of Life: A Longitudinal Study from before and during the Health Crisis. Int. J. Environ. Res. Public Health.

[10] Truzoli R., Pirola V., Conte S. (2021). The Impact of Risk and Protective Factors on Online Teaching Experience in High School Italian Teachers during the COVID-19 Pandemic. J. Comput. Assist. Learn..

[11] Zhao Y., Guo Y., Xiao Y., Zhu R., Sun W., Huang W., Liang D., Tang L., Zhang F., Zhu D. (2020). The Effects of Online Homeschooling on Children, Parents, and Teachers of Grades 1–9 during the COVID-19 Pandemic. Med. Sci. Monit. Int. Med. J. Exp. Clin. Res..

[12] De Araújo L.A., Veloso C.F., de Campos Souza M., de Azevedo J.M.C., Tarro G. (2021). The Potential Impact of the COVID-19 Pandemic on Child Growth and Development: A Systematic Review. J. Pediatr..

[13] Aurini J., Davies S. (2021). COVID-19 School Closures and Educational Achievement Gaps in Canada: Lessons from Ontario Summer Learning Research. Can. Rev. Sociol. Can. Sociol..

[14] Carroll N., Sadowski A., Laila A., Hruska V., Nixon M., Ma D.W.L., Haines J., Study G.F.H. (2020). The Impact of COVID-19 on Health Behavior, Stress, Financial and Food Security among Middle to High Income Canadian Families with Young Children. Nutrients.

[15] Engzell P., Frey A., Verhagen M.D. (2021). Learning Loss Due to School Closures during the COVID-19 Pandemic. Proc. Natl. Acad. Sci. USA.

[16] Gloster A.T., Lamnisos D., Lubenko J., Presti G., Squatrito V., Constantinou M., Nicolaou C., Papacostas S., Aydın G., Chong Y.Y. (2020). Impact of COVID-19 Pandemic on Mental Health: An International Study. PLoS ONE.

[17] Hill R.M., Rufino K., Kurian S., Saxena J., Saxena K., Williams L. (2021). Suicide Ideation and Attempts in a Pediatric Emergency Department before and during COVID-19. Pediatrics.

[18] Tang S., Xiang M., Cheung T., Xiang Y.-T. (2021). Mental Health and Its Correlates among Children and Adolescents during COVID-19 School Closure: The Importance of Parent-Child Discussion. J. Affect. Disord..

[19] Ozamiz-Etxebarria N., Berasategi Santxo N., Idoiaga Mondragon N., Dosil Santamaría M. (2021). The Psychological State of Teachers during the COVID-19 Crisis: The Challenge of Returning to Face-to-Face Teaching. Front. Psychol..

[20] Lindwall M., Gerber M., Jonsdottir I.H., Börjesson M., Ahlborg Jr G. (2014). The Relationships of Change in Physical Activity with Change in Depression, Anxiety, and Burnout: A Longitudinal Study of Swedish Healthcare Workers. Health Psychol..

[21] VanKim N.A., Nelson T.F. (2013). Vigorous Physical Activity, Mental Health, Perceived Stress, and Socializing among College Students. Am. J. Health Promot..

[22] Wunsch K., Kasten N., Fuchs R. (2017). The Effect of Physical Activity on Sleep Quality, Well-Being, and Affect in Academic Stress Periods. Nat. Sci. Sleep.

[23] Alotaibi A.D., Alosaimi F.M., Alajlan A.A., Abdulrahman K.A.B. (2020). The Relationship between Sleep Quality, Stress, and Academic Performance among Medical Students. J. Family Community Med..

[24] Kim H.J., Oh S.Y., Joo J.H., Choi D.-W., Park E.-C. (2019). The Relationship between Sleep Duration and Perceived Stress: Findings from the 2017 Community Health Survey in Korea. Int. J. Environ. Res. Public Health.

[25] Schwarz J., Gerhardsson A., van Leeuwen W., Lekander M., Ericson M., Fischer H., Kecklund G., Åkerstedt T. (2018). Does Sleep Deprivation Increase the Vulnerability to Acute Psychosocial Stress in Young and Older Adults?. Psychoneuroendocrinology.

[26] Pensuksan W.C., Lertmaharit S., Lohsoonthorn V., Rattananupong T., Sonkprasert T., Gelaye B., Williams M.A. (2016). Relationship between Poor Sleep Quality and Psychological Problems among Undergraduate Students in the Southern Thailand. Walailak J. Sci. Technol..

[27] Vogel E.A., Zhang J.S., Peng K., Heaney C.A., Lu Y., Lounsbury D., Hsing A.W., Prochaska J.J. (2022). Physical Activity and Stress Management during COVID-19: A Longitudinal Survey Study. Psychol. Health.

[28] Moriarty T., Bourbeau K., Fontana F., McNamara S., Pereira da Silva M. (2021). The Relationship between Psychological Stress and Healthy Lifestyle Behaviors during COVID-19 among Students in a US Midwest University. Int. J. Environ. Res. Public Health.

[29] Kredlow M.A., Capozzoli M.C., Hearon B.A., Calkins A.W., Otto M.W. (2015). The Effects of Physical Activity on Sleep: A Meta-Analytic Review. J. Behav. Med..

[30] Baron K.G., Reid K.J., Zee P.C. (2013). Exercise to Improve Sleep in Insomnia: Exploration of the Bidirectional Effects. J. Clin. Sleep Med..

[31] US Department of Education, Office of Postsecondary Education Enrollment in Teacher Preparation Programs. *Title II News You Can Use*
**2015**. https://eric.ed.gov/?id=ED576131.

[32] Cohen S., Spacapan S., Oskamp S. (1988). Perceived Stress in a Probability Sample of the United States. The Social Psychology of Health.

[33] Cohen S., Kamarck T., Mermelstein R. (1994). Perceived Stress Scale. Meas. Stress A Guid. Health Soc. Sci..

[34] Craig C.L., Marshall A.L., Sjöström M., Bauman A.E., Booth M.L., Ainsworth B.E., Pratt M., Ekelund U.L.F., Yngve A., Sallis J.F. (2003). International Physical Activity Questionnaire: 12-Country Reliability and Validity. Med. Sci. Sport. Exerc..

[35] Dinger M.K., Behrens T.K., Han J.L. (2006). Validity and Reliability of the International Physical Activity Questionnaire in College Students. Am. J. Health Educ..

[36] Famodu O.A., Barr M.L., Holásková I., Zhou W., Morrell J.S., Colby S.E., Olfert M.D. (2018). Shortening of the Pittsburgh Sleep Quality Index Survey Using Factor Analysis. Sleep Disord..

[37] Buysse D.J., Reynolds III C.F., Monk T.H., Berman S.R., Kupfer D.J. (1989). The Pittsburgh Sleep Quality Index: A New Instrument for Psychiatric Practice and Research. Psychiatry Res..

[38] Werneck A.O., Silva D.R., Malta D.C., Lima M.G., Souza-Júnior P.R.B., Azevedo L.O., Barros M.B.A., Szwarcwald C.L. (2020). The Mediation Role of Sleep Quality in the Association between the Incidence of Unhealthy Movement Behaviors during the COVID-19 Quarantine and Mental Health. Sleep Med..

[39] Gluschkoff K., Elovainio M., Kinnunen U., Mullola S., Hintsanen M., Keltikangas-Järvinen L., Hintsa T. (2016). Work Stress, Poor Recovery and Burnout in Teachers. Occup. Med..

[40] Madden S.K., Cordon E.L., Bailey C., Skouteris H., Ahuja K., Hills A.P., Hill B. (2020). The Effect of Workplace Lifestyle Programmes on Diet, Physical Activity, and Weight-Related Outcomes for Working Women: A Systematic Review Using the TIDieR Checklist. Obes. Rev..

[41] Swider B.W., Zimmerman R.D. (2010). Born to Burnout: A Meta-Analytic Path Model of Personality, Job Burnout, and Work Outcomes. J. Vocat. Behav..

